# First outbreaks and phylogenetic analyses of avian influenza H9N2 viruses isolated from poultry flocks in Morocco

**DOI:** 10.1186/s12985-016-0596-1

**Published:** 2016-08-15

**Authors:** Mohammed EL Houadfi, Siham Fellahi, Saadia Nassik, Jean-Luc Guérin, Mariette F. Ducatez

**Affiliations:** 1 Unité de Pathologie Aviaire, Institut Agronomique et Vétérinaire Hassan II, Rabat, Morocco 10000; 2 IHAP, Université de Toulouse, INRA, ENVT, F-31076 Toulouse, France; 3 Avian Pathology Unit, Department of Pathology and Veterinary Public Health, Agronomy and Veterinary Institute Hassan II, BP 6202, Rabat- Instituts, Rabat, Morocco

**Keywords:** Influenza Virus, H9N2, Phylogenetic analyses, Morocco

## Abstract

**Background:**

H9N2 avian influenza viruses continue to spread in poultry and wild birds worldwide. Morocco just faced its first H9N2 influenza virus outbreaks early 2016 affecting different types of poultry production. After its introduction, the virus spread very rapidly throughout the country.

**Methods:**

Samples were collected from 11 chicken flocks with high morbidity and mortality rates. Four viruses were successfully isolated from broiler chickens and one from broiler breeders and fully sequenced.

**Results:**

Phylogenetic and molecular markers analyses showed the Moroccan viruses belonged to the G1 lineage and likely originated from the Middle East. As known for H9N2 viruses, the Moroccanisolates possess several genetic markers that enhance virulence in poultry and transmission to humans.

**Conclusion:**

The present study demonstrated that under field conditions H9N2 could have a devastating effect on egg production and mortalities and highlighted a lack of surveillance data on the pathogen in the region.

## Background

The influenza A virus (IAV) H9N2 is of great economical concern for poultry production and public health [1]. IAV H9N2 is now enzootic in many parts of the world. In chickens, the first report dates from 1992 in China [2, 3] and the virus was reported in South Korea in 1996 [4] and Iran and Pakistan in 1998 and 1999 respectively [5, 6]. The virus spread in the early 2000s to several countries of the Middle East, including Israel, Saudi Arabia, United Arab Emirates and Sultanate of Oman [7–9]. Then, the virus appeared in North African countries: Egypt, Libya and Tunisia [10, 11].

Despite the fact that H9N2 is considered as low pathogenic virus, it has been reported that several microbial agents of the respiratory tract and some environmental factors exacerbate IAV H9N2 infections, leading to very severe respiratory disease and causing mortality up to 65 % in broiler chickens and up to 70 % drop in egg production in layers and breeders [12, 13]. It has also been reported that IAV H9N2 affected many species of domesticated and wild birds such as falcon, partridge, quail, houbara, pigeon, sparrow and other species. In Pakistan, sparrows were found to play a very important role in the transmission of the virus between farms [14]. IAV H9N2 was detected in mammalian species such as dogs and cats [15]. In affected countries, high seroprevalences to H9N2 virus were detected in workers of poultry farms and poultry processing plants [16]. The virus itself has been isolated from Human and is monitored continuously by the World Health Organization (WHO).

Molecular analyses of H9N2 viruses isolated during the last two decades revealed that these viruses evolve fast and represent a genetically diverse population [17]. H9N2 influenza viruses circulate in poultry and wild birds worldwide and cluster into two main lineages in Eurasia: A/quail/Hong Kong/G1/97-like viruses (G1-like) and A/duck/Hong Kong/Y280/97-like viruses (Y280-like) [18].

In Morocco, low pathogenic avian influenza subtype H9N2 virus was first diagnosed in poultry in January 2016. As an initial response, the National Sanitary and Security Food Office of Morocco authorized emergency vaccinations of all types of poultry production. This study describes the first report and rapid spread of IAV H9N2 to all areas of the country within a few weeks and its devastating effects in different types of poultry productions. Genetic analyses of Moroccan H9N2 isolates will be presented.

## Methods

### Case history

11 poultry flocks from different regions of Morocco showing very high mortality, decrease in feed consumption and very severe respiratory signs including sneezing, coughing, rales and gasping were analysed by real time RT-PCR for the presence of nucleic acids of infectious bronchitis, Newcastle disease, virulent infectious bursal disease viruses and IAV [10, 19–21]. Details on the flocks sampled in this study are presented in Table 1.

**Table 1:** Detection and isolation of Moroccan H9N2 viruses from different types of birds and areas of Morocco in 2016

Sample number	Region of Morocco	Month of sample collection	Type of birds	Age	AIV detection RT-PCR Ct Value	Virus isolation	Full genome sequencing
1	Kenitra	January	Broiler	31 days	18,9	yes	yes
2	Fes	January	Breeders	43 weeks	20,6	yes	yes
3	Oujda	February	Broiler	27 days	23,2	yes	yes
4	Temara	January	Broiler	22 days	37,2	-	-
5	Tiznit	February	Broiler	33 days	29,5	-	-
6	Meknes	January	Broiler	30 days	33,1	-	-
7	Casablanca	February	Broiler	47 days	17,5	yes	yes
8	Sefrou	January	Broiler	32 days	negative	-	-
9	Meknes	January	Broiler	39 days	32,4	-	-
10	Oujda	January	Broilers	36 days	29,6	-	-
11	EL Hajeb	January	Broiler	19 days	23,1	yes	yes

### Virus isolation

Five samples out of 11 with low Ct values in real time RT-PCR for IAV were selected for inoculation into the allantoic cavities of 9–day–old specific pathogen free chickens embryos, incubated for 48 h at 37 °C then chilled at 4 °C for 4 h before harvesting. The allantoic fluid was harvested, clarified, tested by real time RT-PCR for IAV genome, and then stored at −80 °C until use.

### Full genome amplification and sequencing of H9N2 Moroccan isolates

PCR amplification was performed in a thermocycler (GeneAmp PCR System 9700, Applied Biosystems) using segment specific primers described previously by Hoffman et al., (2001) [22] and the One step RT-PCR kit (Qiagen) following instructions of the manufacturer. Reactions were performed according to the following protocol: 50 °C for 30 min and 95 °C for 5 min, followed by 40 cycles of 95 °C for 30 s, 54 °C to 57 °C for 30 s, 72 °C for 1 min30 s, and a final elongation step of 6 min at 72 °C. PCR products of the expected length were purified with the Nucleospin gel and PCR cleanup kit (Macherey Nagel) according to the manufacturer’s instructions. Sanger sequencing method was carried out on all the segments using the same primers as used for the RT-PCR. Sequencing was performed on a 3130XL Applied Biosystems capillary sequencer at the Plateau de Génomique GeT-Purpan, UDEAR UMR 5165 CNRS/UPS, CHU PURPAN, Toulouse, France.

### Sequences and phylogenetic analyses

Complete sequence data of five H9N2 isolates were manually assembled using BioEdit software package version 5.0.9 [23]. The open source BLAST program (National Center for Biotechnology Information, Bethesda MD, http://blast.ncbi.nlm.nih.gov/Blast.cgi) was used for sequence comparison. The best fitting nucleotide substitution model was estimated by means of hierarchical likelihood ratio approach using Mega 6.06 [24] for each sequences alignment. Phylogenetic analysis and tree construction were thus generated using the maximum likelihood method, General Time Reversible model, with 500 bootstrap replicates with Mega 6.06, and bootstrap values above 50 were labelled on major tree branches. Sequences were submitted to the EMBL/GenBank databases.

## Results

### Epidemiology of H9N2 outbreaks in Morocco

Up to the end of December 2015, no official report on the presence of any subtype of avian influenza in commercial poultry farms or backyard flocks had been recorded in Morocco. During the second week of January 2016, 11cases of chickens from different poultry production areas around the cities of Fez, Meknes, Kenitra and Rabat, were received for diagnosis purpose in our laboratory (Table 1). Most cases showed very high decrease in feed consumption and very severe respiratory signs (sneezing, coughing, rales and gasping). All cases showed a very rapid increase in mortality rates (2 % to 10 % per day between the 3rd and the 6th day after onset of disease). All affected flocks were over three weeks of age and some flocks were at the selling period. In a few cases, diarrhoea was reported. All flocks were vaccinated, as routinely performed, against Newcastle, infectious bronchitis and Gumboro diseases. By mid-February, all parts of the country were affected and the disease spread to layers and breeders farms, which showed very severe respiratory signs, mortality rates ranging from 2 to 15 %and drastic drop in egg production (up to 80 %) with no complete recovery after several weeks. Indeed, up to 10 weeks after the infection, egg production remained between 20 and 25 % lower than normal levels. Turkey flocks were similarly affected at different ages, with similar clinical signs as seen in chickens as well as sinusitis with facial swellings, and mortality rates around 10 %, but with a large inter-flock variability, likely due to other contributing factors.

### Post-mortem examinations

**Fig. 1:**
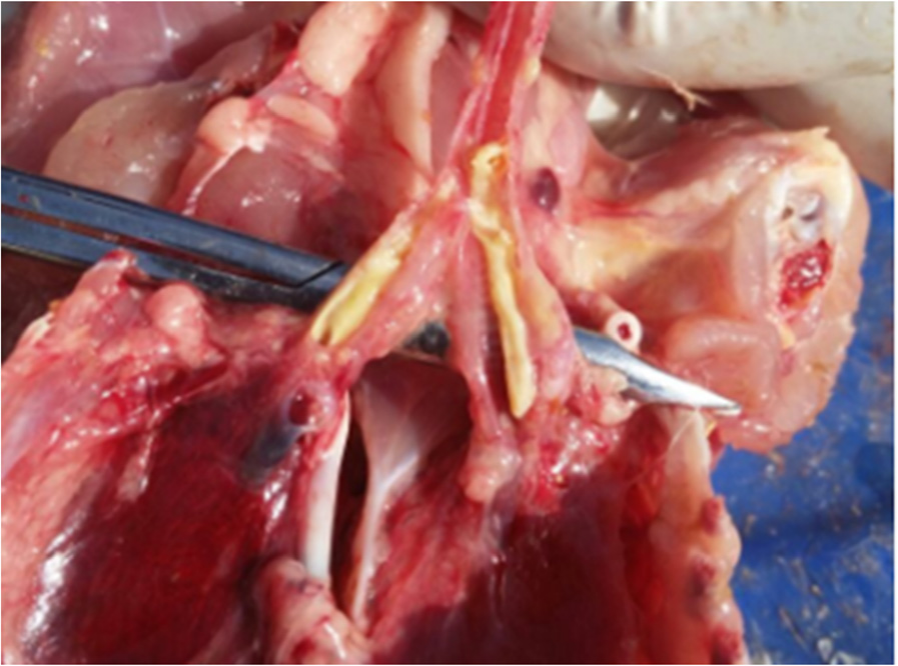
Congested trachea with fibrinous casts in both bronchi. Picture taken from a Moroccan H9N2 outbreak in January 2016, from a broiler chicken at 31 days of age

Post mortem examinations revealed that all carcasses were congested, some showing oculonasal discharge and infraorbital sinusitis. The main consistent lesions were congested or haemorrhagic tracheas with fibrinous exudates in the lumen and presence of fibrinous casts in bronchi (Fig. 1), haemorrhagic lungs, pneumonia with fibrinous exudates and airsaculitis. In some cases congested and swollen kidneys, spleen and liver were observed.

### Real time RT-PCR

Eleven samples from respiratory tissues collected from different cases and analysed by real time RT-PCR for the presence of Infectious bronchitis, Newcastle disease and virulent IBD viruses werenegative. However10 out of 11 samples were positive for IAV with cycle threshold (Ct) values ranging from 17.5 to 35.1 (Table 1).

### Phylogenetic analyses

**Fig. 2:**
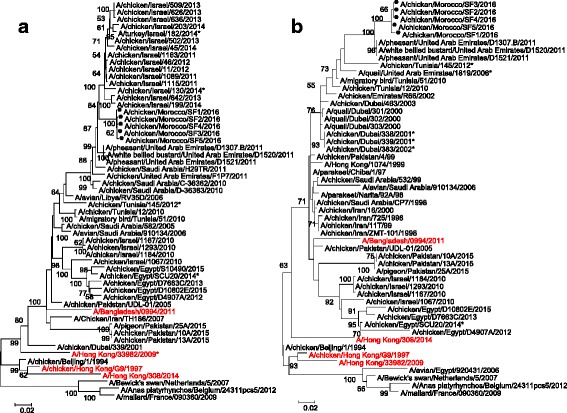
Phylogenetic analysis of Moroccan HA (A) and NA (B) gene segments. The nucleotide sequences of Moroccan H9N2 viruses characterized in our study (with black circle shaped symbols) were compared with relevant virus sequences available in GenBank and GISAID databases. In brief, we selected the first 20 hits by BLAST search, included WHO recommended vaccine strains (in red font), reference viruses, and relevant sequences from neighbouring areas

**Fig. 3:**
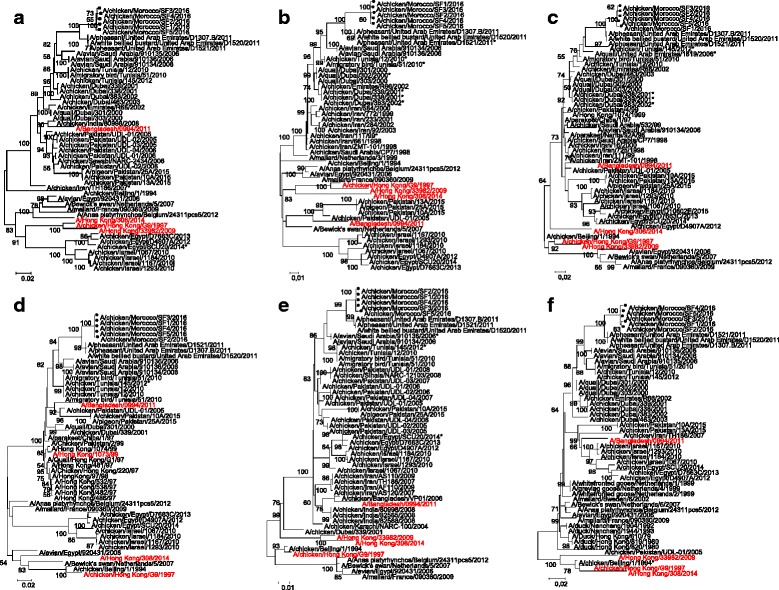
Phylogenetic analysis of Moroccan PB2 (A), PB1 (B), PA (C), NP (D), M (E), and NS (F) gene segments. The nucleotide sequences of Moroccan H9N2 viruses characterized in our study (with black circle shaped symbols) were compared with relevant virus sequences available in GenBank and GISAID databases. In brief, we selected the first 20 hits by BLAST search, included WHO recommended vaccine strains (in red font), reference viruses, and relevant sequences from neighbouring areas

The phylogenetic analyses showed that the Moroccan H9N2 viruses of broiler and breeder broiler origins were of the G1 lineage and closely related to each other (Fig. 2). The nucleotide sequence identities among the five Moroccan strains ranged from 98 to 100 %. Genes segments coding for polymerase basic 2 (PB2), polymerase basic 1 (PB1), acidic polymerase (PA), hemagglutinine (HA), nucleoprotein (NP), neuraminidase (NA), matrix (M) and non structural (NS) proteins all clustered with G1 lineage viruses, closely related to an A/pheasant/United Arab Emirates/D1521/2011-like H9N2 virus, as indicated by the trees topology (Figs. 2, 3) and by the high nucleotide sequence identity: 96.1 to 98.2 %.

### Molecular characterization

#### Hemagglutinin cleavage site and receptor binding domains

The HA of Moroccan H9N2 isolates analyzed in this study had a RSSR*GLF motif at the HA0 cleavage site, a characteristic of the LPAI viruses in poultry. The HA proteins harboured seven potential glycosylation sites with one additional glycosylation site (residue 82) as compared to recently characterized Egyptian H9N2 viruses (Table 2). All the Moroccan viruses had 158 N, H183, 226 L and 391 K substitutions in their receptor binding site (H3 numbering) known to confer preference to humanlike receptors [25–27].

**Table 2:** Molecular determinants of virulence, host specificity and drug resistance in Moroccan H9N2 isolates

Protein	Molecular determinants of virulence	Molecular determinants of host specificity (adaptation to mammals)	Molecular determinants of drug resistance	Miscellaneous
PB2	147 V, 504 V	318R, 590S, 661 T		
PB1		13P		
PB1-F2	66 N	82 L		Truncated protein of 52 aa
PA	127 V, L550, 672 L	I100, R312, 409 N		
PA-X				Full length 252 amino acids
HA^a^		158 N, 183H, 226 L, 391 K		Cleavage site: RSSRGL*F Glycosylation sites: 29NSTE, 82NPSC, 105NGTC, 141NVTY, 298 NSTM, 305NISK, 492NGTY
NP		372D		
NA			None	No stalk deletion
M1		15I		
M2		D16	31 N (amantadane resistance)	
NS1	42S, 189D			No 80–84 deletion and GSEV PL motif
NS2	31 M, 56H			

^a^H3 numbering

#### NA structural features

The NA sequences of the five Moroccan isolates had a nucleotide sequence identity between 99.8 and 100 % when compared to each other. The deduced NA amino acid sequence did not have any previously identified mammalian-adaptation associated substitutions and no stalk deletion (Table 2).

#### Molecular features of internal genes

Three mammalian adaptation markers were identified in Moroccan PB2 proteins: 318R, 590S and 661 T [28–30]. An additional substitution, 504 V, was observed in all 5 isolates. A unique molecular determinant of host specificity described as avian–human signature 13Pwas observed for PB1 [30]. A truncated 52 amino acids PB1-F2 protein was harbored by the 5 Moroccan isolates. The PA proteins had three amino acid substitutions associated within creased virulence: 127 V, 550 L and 672 L as previously reported by Kandeil et al., 2014, Rolling et al., 2009 and Chen et al., 2006 [27, 31, 32]. All Moroccan isolates had 409 N, 100I and 312R substitutions, which are associated with mammalian adaptation [28]. The deduced NP amino acid sequence had 372D substitution previously identified as important for changing host range from avian to human [30]. On the M2 protein, the 10 L residue, marker of the G1 lineage, was conserved. All Moroccan isolates also harbored as substitution at M2 amino acid position 31:S31N, suggesting a resistance to the amantadane [8, 30]. None of the Moroccan isolates contained deletion at amino acid positions 80–84 in NS1. Moreover, all Moroccan isolates possessed the 42S and 189D virulence markers and the NS1 GSEV motif in NS1 (Table 2). The molecular characterization of NS2 protein revealed the presence of 31 M and 56H markers: two known virulence determinants.

## Discussion

This study illustrates clearly how fast the H9N2 IAV did spread to the whole country within a very short time likely through the movement of vehicles transporting live birds to live bird markets (over 70 % of the poultry trade in Morocco) and feed delivery vehicles as previously reported in other countries in the world [27, 30]. According to Wernery et al., (2013) smuggled H9N2-infected falconry birds may contribute to the spread of these viruses to wild birds, domestic poultry, and humans [30].

It was important to notice that common biosecurity measures applied routinely in breeder and layer production sites were insufficient to prevent the entry of virus. Although H9N2 viruses are considered as low pathogenic, the recent Moroccan situation showed that it could cause indeed very heavy losses in different types of poultry production systems. In order to limit disease spread to the other parts of the country, several measures were quickly implemented including the importation of inactivated H9N2 vaccine to immunise all types of poultry productions. H9N2 viruses are prevalent in terrestrial poultry throughout Asia and have been isolated from poultry outbreaks worldwide. In addition to the economic losses related to drop in egg production and mortalities in different type of poultry flocks, the zoonotic potential of the pathogen should not be under estimated [2]. In fact, H9N2 viruses infect both avian and mammalian species and might constitute significant donors of genetic material to emerging human type A influenza viruses [33, 34]. It was reported that the natural isolates of H9N2 viruses were transmissible among ferrets via respiratory droplets, emphasizing the pandemic potential of H9N2 viruses [35] and human cases of H9N2 infections have been reported by WHO [36].

In the present study our findings clearly demonstrate a relationship between viruses that recently emerged in Morocco and viruses isolated in the Middle East of the G1 lineage. In order to evaluate the genetic similarity of the Moroccan and Middle Eastern viruses, phylogenetic and genetic identities were calculated for each gene segment. The limited influenza virus sequence data available from North African and Middle-Eastern countries makes the identification of the origin of Moroccan H9N2 virus outbreaks very difficult. The similarities between our viruses and those from the United Arab Emirates and Israel confirm that the viruses isolated from Morocco in 2016 are the likely direct progenitors of those circulating in the Middle East [9, 27, 30, 37]. On the HA phylogeny (Fig. 2), Moroccan viruses indeed clustered between 2011 United Arab Emirates and 2011–2014 Israeli viruses. For the other 7 gene segments, the Moroccan isolates sequences formed a distinct cluster with pheasant and white-bellied bustard viruses isolated in the United Arab Emirates (supported by high bootstrap values on all trees, over 99 %, Figs. 2 and 3). However only HA sequences are available for the Israeli viruses and the most recent Emirati viruses sequences are from 2011: the clear lack of surveillance data in the region may considerably bias the analysis.

Molecular characterization of the HA protein of Moroccan isolates revealed one addition potential glycosylation site as compared to recent Egyptian H9N2 HAs. Previous studies showed a link between additional glycosylation sites on HA and the adaptation of H9N2 in poultry [38]. The presence of Q226L mutation in HA is known to enable binding to human-like receptors [39]. H9N2 viruses in Morocco possess several genetic markers shown to enhance virulence in poultry and enable transmission to humans. Previously, several studies demonstrated that G1-like H9N2 viruses were the likely donors of the six internal genes of the H5N1 viruses causing the bird flu outbreak in humans in 1997 in Hong Kong [40]. Moreover, H9N2 influenza viruses continue to reassort with circulating H7N9 viruses, resulting in multiple genotypes of H7N9 viruses [2, 34, 41]. Full genome sequencing of H9N2 influenza viruses is therefore essential to better predict putative zoonotic transmissions. How the disease was introduced to the country is still not understood and several investigations are currently in progress.

## Conclusion

The present study investigated the first introduction and isolation of avian H9N2 in Morocco. Although H9N2 viruses are considered as low pathogenic, the recent Moroccan situation showed that it could cause indeed very heavy losses in different types of poultry production. Phylogenetic analyses showed that the Moroccan viruses belonged to the G1 lineage and likely originated from the Middle East. How the disease was introduced to the country is still not understood.

## Abbreviations

HA, hemagglutinine; IAV, Influenza A virus; M, matrix; NA, neuraminidase; NP, nucleoprotein; NS, non structural; PA, acidic polymerase; PB1, polymerase basic 1; PB2, polymerase basic 2; RT-PCR, reverse transcription polymerase chain reaction; WHO, World Health Organization
